# Early results of a novel technique for anterior vaginal wall prolapse repair: anterior vaginal wall darn

**DOI:** 10.1186/1471-2490-14-51

**Published:** 2014-06-29

**Authors:** Osman Köse, Hasan S Sağlam, Şükrü Kumsar, Salih Budak, Hüseyin Aydemir, Öztuğ Adsan

**Affiliations:** 1 Department of Urology, Faculty of Medicine, Sakarya University and Training and Research Hospital, 54100 Sakarya, Turkey; 2 Beyaz Kent Sitesi, Beşköprü M. Girne C., 54100 Sakarya, Turkey

**Keywords:** Anterior vaginal wall prolapse, Darn, Pelvic organ prolapse, Stress urinary incontinence, Surgical technique

## Abstract

**Background:**

The aim of this study was to describe the results of a 1-year patient follow-up after anterior vaginal wall darn, a novel technique for the repair of anterior vaginal wall prolapse.

**Methods:**

Fifty-five patients with anterior vaginal wall prolapse underwent anterior vaginal wall darn. The anterior vaginal wall was detached using sharp and blunt dissection via an incision beginning 1 cm proximal to the external meatus and extending to the vaginal apex. The space between the tissues that attach the lateral vaginal walls to the arcus tendineus fasciae pelvis was then darned. Cough Stress Test, Pelvic Organ Prolapse Quantification, seven-item Incontinence Impact Questionnaire, and six-item Urogenital Distress Inventory scores were performed 1-year postoperatively to evaluate recovery.

**Results:**

One-year postoperatively, all patients were satisfied with the results of the procedure. No patient had vaginal mucosal erosion or any other complication.

**Conclusions:**

One-year postoperative findings for patients in this series indicate that patients with stage II–III anterior vaginal wall prolapse were successfully treated with the anterior vaginal wall darn technique.

## Background

Pelvic organ prolapse (POP), a condition characterized by a downward descent of the pelvic organs, causing the vagina to protrude, afflicts millions of women worldwide and is increasingly recognized as a global burden on women’s health [1]. The social, psychological, and economical cost of POP can be high [2,3]. During their lifetimes, nearly 10% of women will require surgery for POP, urinary incontinence, or both. Of these, 30% will undergo two or more surgical procedures, presenting a challenge to gynaecologist and urologist [4]. The anterior vaginal wall is the segment of the vagina that most commonly prolapses and is most likely to fail long term after surgical correction [5]. Central defects have traditionally been treated with anterior colporrhaphy, which entails central plication of the fibromuscular layer of the anterior vaginal wall [6]. Paravaginal defects have been repaired with vaginal paravaginal repair. Combination vaginal repair of these defects has been performed with these two operations [7]. Recurrent anterior vaginal wall prolapse following conventional repair has been reported in more than 30% of cases [8].

In an effort to improve outcomes of transvaginal prolapse repair, a number of graft materials have been introduced to complement, reinforce, or replace native tissue in reconstructive surgical procedures. Although abdominal sacrocolpopexy and suburethral sling procedures, the standard of care, have been shown to be effective, there is considerable debate over the use of permanent mesh and biologic grafts for transvaginal POP repair [9,10].

The use of synthetic graft material for the repair of anterior vaginal wall prolapse has been limited by potential complications related to the mesh, including mesh erosion and contraction, dyspareunia, pelvic pain and infection. A lack of comparative data and an anticipated incidence of graft-related complications such as graft erosion and infection have caused debate among surgeons regarding the use of synthetic grafts [11].

For this reason, we introduce a new technique, anterior vaginal wall darn (AVWD) which is carried out without mesh. Unlike colporrhaphy, this technique does not cause tissue tension and is easy to perform, and in contrast to the use of mesh, it does not corrupt the anatomical structures.

## Methods

Fifty-five patients who had been experiencing POP symptoms for the previous 9 months and stage II–III prolapse of the anterior vaginal wall were enrolled between September 2011 and July 2012. Patients provided written informed consent to participate, and the study protocol had been approved by the Ethics Committee of Sakarya University Medical Faculty.

Preoperative evaluation consisted of complete medical history, gynaecological examination, cough stress test (CST), voiding diary, daily pad use, Q-tip test, and seven-item Incontinence Impact Questionnaire (IIQ-7) and six-item Urogenital Distress Inventory (UDI-6) scores. Patients’ symptoms were also evaluated via standard questions asked by the examining physician. The severity of prolapse was assessed using the POP Quantification (POPQ) system adopted by the International Continence Society. Daily pad weight was used to quantify patients’ subjective complaints.

**Figure 1:**
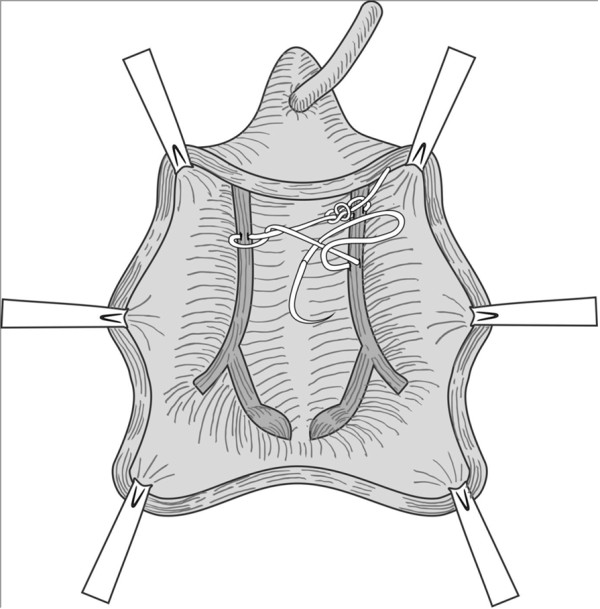
Bites were taken from alternate sides of the arcus tendineus fasciae pelvis using continuous locking 2–0 polypropylene suture, and the tissue edges were slightly approximated.

**Figure 2:**
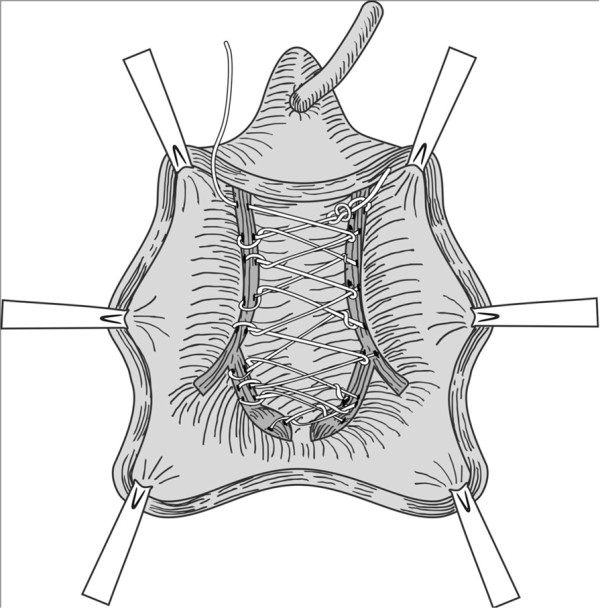
The suture was extended continuously to the distal aspect to form a darn.

**Figure 3:**
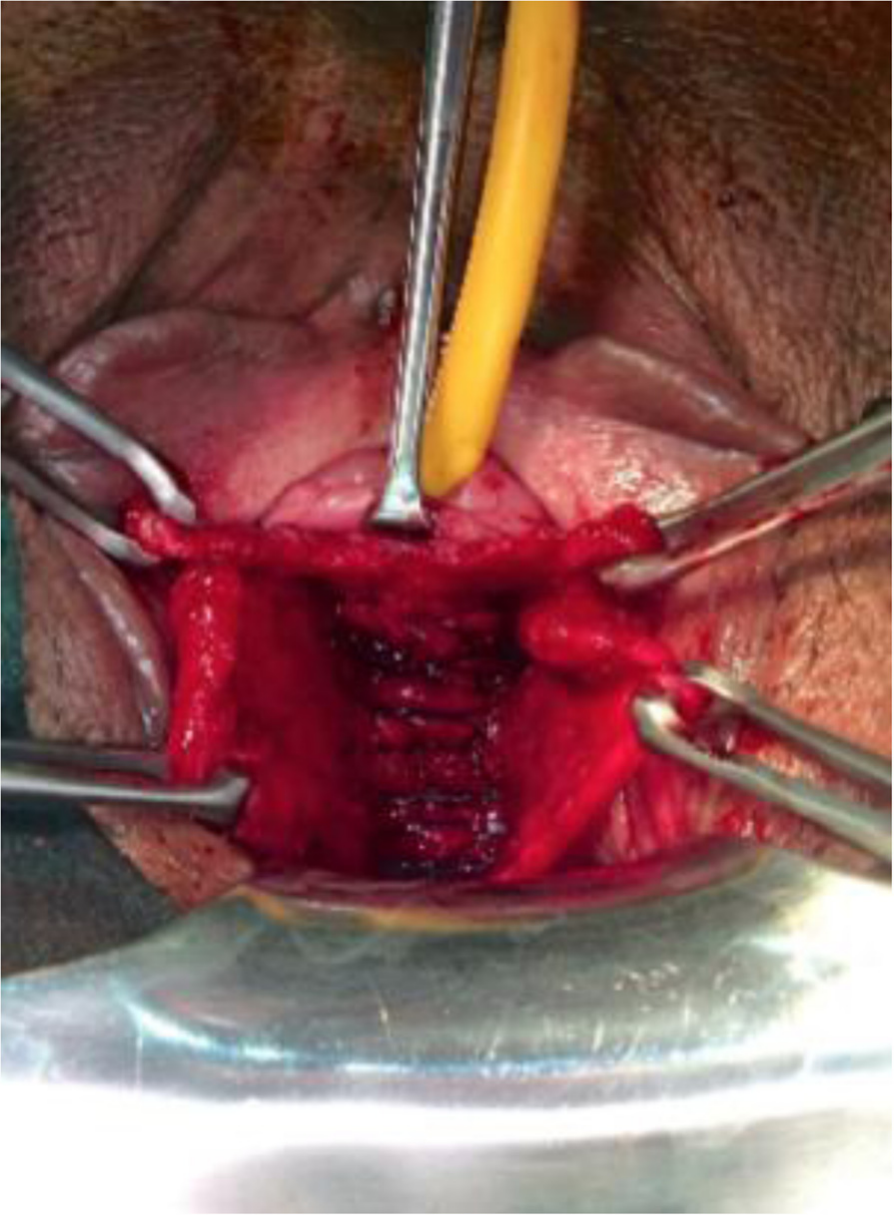
**Surgical view of the ends of the suture.** (Consent was obtained from the patient to publish this image. A copy of the written consent is available for review by the Editor of this journal).

Exclusion criteria were a history of pelvic or vaginal surgery, predominant urge incontinence, pelvic or systemic infection, inguinal or vulvar abscess, pregnancy, urinary tract obstruction or renal insufficiency, pelvic pain unrelated to prolapse, vaginal bleeding of unknown aetiology, blood coagulation disorders, pelvic malignancy or previous irradiation of the pelvic region, vaginal erosion or severe vaginal atrophy, vaginal or urethral fistula, and known allergy to the suture material. Patients requiring concomitant vaginal vault suspension, such as sacrospinous ligament fixation; sacrocolpopexy for vaginal prolapse or uterine procidentia; or laparotomy or laparoscopy for any reason, were also excluded.All AVWD procedures were performed by the same surgeon as follows: after insertion of an 18-Fr indwelling urethral catheter, an adequate volume of normal saline was injected under the vaginal mucosa to provide comfortable dissection in an accurate plane with limited haemorrhage. A midline incision was made beginning 1 cm proximal to the external urethral meatus and extending to the vaginal apex. The anterior vaginal wall was detached from the urinary bladder beyond the anterior vaginal sulcus using sharp and blunt dissection until the arcus tendinous fasciae pelvis (ATFP) was exposed. Using a continuous locking 2–0 polypropylene suture, bites were taken on alternate sides from distal to proximal in the ATFP and the tissues that attach the lateral walls of the vagina to the ATFP (Figure 1). After 6 cm, at the point where the ATFP exits the anterior vaginal wall, the sutures were placed medial to the perivesical fascia for 2–3 cm. The running sutures were turned back from the cardinal ligaments without being tied and were extended continuously to the distal aspect to form a darn. The suture ends were tied together (Figures 2 and 3). The traumatized vaginal mucosa was trimmed and closed with a continuous absorbable sutures, and a vaginal pack soaked in antibiotic solution was inserted.

Postoperative evaluation, including POPQ measurement, UDI-6, and IIQ-7 scores, was performed for each patient, 1 year after the AVWD procedure. A Q-tip test was performed to evaluate urethral hypermobility. Pre- and postoperative questionnaire scores and POPQ measurements were analyzed using Wilcoxon signed rank test. SPSS for Windows, Version 12.0 (SPSS, Inc., Chicago, IL, USA) was used for data analysis. The level of statistical significance was set at P < 0.05.

## Results

Fifty-five patients with anterior POP stage II–III were eligible to participate in the study. The patients age range was 35–67 years (median age 51 years). Baseline demographic and clinical parameters are shown in Table 1. Median surgical duration was 40 minutes (range: 30–45 min), mean duration of hospitalization was 1.7 days (range: 1–2 days), and the average time to void was 1.4 days (range: 1–2 days). Pre- and postoperative POPQ measurements, shown in Table 2, reveal significant improvements at points Aa and Ba. Similarly, UDI-6 and IIQ-7 scores were significantly lower postoperatively (P < 0.001) (Table 2). Similarly, UDI-6 and IIQ-7 scores were significantly lower postoperatively (p < 0.001) (Table 2).

**Table 1:** Patient characteristics at baseline

**Characteristic**	**n = 55 (%)**
Age	51 ± 16.3
Body mass index, kg/m^2^	
<30	36 (65.45)
30-40	12 (21.81)
>40	7 (12.72)
Parity	3 (0–6)
Topical oestrogen	13 (23.6)
Hypertension	15 (37.2)
Smoking status	
Current	10 (18.1)
Former	13 (23.6)

**Table 2:** POPQ and incontinence-related quality values

	**Before operation**	**After operation**	**P value**
POPQ measurements Aa (cm)	1.6 ± 1.0	−2.1 ± 0.7	<0.001
POPQ measurements Ba (cm)	2.3 ± 1.5	−2.0 ± 0.8	<0.001
POPQ measurements Ap (cm)	−2.2 ± 0.6	−2 ± 0.7	0.17
POPQ measurements Bp (cm)	−2.78 ± 0.4	−2.61 ± 0.52	0.18
POPQ measurements TVL (cm)	7.62-0.51-	7.13-0.62	0.53
POPQ measurements C (cm)	−5.5 ± 1.4	−6.3 ± 1.2	0.046
UDI-6	8.9 ± 3.7	1.6 ± 1.2	<0.001
IIQ-7	10.5 ± 5.3	0.9 ± 0.8	<0.001
Q-TT	28.7 ± 6.2	15.3 ± 10.4	<0.018
Pad count (d)	4.3 ± 1.6	0.4 ± 0.9	<0.001
Residual urine volume (ml)	58.1 ± 13.2	32.2 ± 10.6	0.024

IIQ-7, seven-item Incontinence Impact Questionnaire; POPQ, Pelvic Organ Prolapse Quantification; Q-TT, Q-Tip test; UDI-6, six-item Urogenital Distress Inventory.

Moderate groin discomfort was the most common complaint immediately postoperatively but disappeared within 10 days of analgesic therapy. One-year postoperatively, all patients underwent a complete evaluation. Symptom relief 12 months post-surgery is shown at Table 3. Upon examination, CST was negative in 90.9% of patients and vaginal examination were appeared normal in all patients. Bladder ultrasonography demonstrated no significant post-void residual urine volume.

**Table 3:** Postoperative symptom relief

	**Preoperative**	**Postoperative**
Pelvic pressure	33	4
Sensation of a mass bulging into the vagina	43	1
Stress urinary incontinence	16	2
Coital incontinence	11	1
Difficulties in emptying the bladder	10	1
Mixed urinary incontinence	13	3
Dyspareunia	12	3

## Discussion

The goal of treatment of POP is to improve patient quality of life rather than prolong survival; therefore, when choosing a surgical method for anterior vaginal wall prolapse, it is important to consider all possible complications as well as treatment outcome [12]. Although conservative treatment is a reasonable initial approach for urinary incontinence, surgical management is usually required for symptomatic grade II-III vaginal prolapse. Many surgical methods are currently known, but unfortunately none can solve the problems caused by POP.

There is a lack of consensus concerning when, where, and how to perform surgery, preferably as a single procedure, to provide the best outcome in patients with POP. When selecting a surgical procedure for POP, pertinent factors, including history of anti-incontinence surgery, sexual activity, coital incontinence, obesity, chronic increases in intra-abdominal pressure, mixed incontinence and concurrent overactive bladder must be considered.

In an effort to improve the outcomes of transvaginal prolapse repair, a number of biologic and synthetic graft materials have been introduced since 1996 for use during reconstructive surgical procedures to reinforce or replace native tissue [13]. Results have been favourable, with anatomical success rates in the range of 59% to 94%; however, the use of mesh in vaginal repair procedures remains controversial [9,14-16]. Recently, significant problems associated with mesh use in vaginal prolapse surgery (dyspareunia, vaginal pain, mesh shrinkage, bladder erosion, fistula, mesh exposure and infection) have been reported [17-21]. Vaginal mesh erosion is one of the most common complications of introducing synthetic material via the vaginal route. Differences in mesh types, follow-up periods, and definitions of success and failure have contributed to inconsistent reported erosion rates. No generally accepted “*safety time zone*” for mesh exposure or erosion has been accepted, and the complication can occur many years after mesh placement. Young age and sexual activity are additional risk factors for mesh exposure [22].

Although there is increasing industry pressure on surgeons to adopt mesh-augmented repairs into their practice and many surgeons are employing the therapy liberally, health organizations such as the US Food and Drug Administration warn urogynaecologists and patients about the dangers of using mesh materials for the treatment of POP [23,24]. The committee for POP at the 3rd International Consultation on Incontinence concluded that there were insufficient data to reach a definitive conclusion regarding the role of biologic or synthetic prosthetic materials in surgical procedures for primary or recurrent prolapse [25].

The available data concerning the results of prolapse surgery remain mixed. Success rate varies substantially depending on the technique used. Despite high anatomical recurrence rates, traditional anterior colporrhaphy, and paravaginal repair have been used for years for the treatment of combined anterior vaginal wall prolapse [26,27]. However, although pubocervical fascia is used to place plication sutures during anterior colporrhaphy, histologic examination of the anterior vaginal wall has failed to show a separate layer of fascia between the vagina and bladder [28], and paravaginal repair has been used only for paravaginal defects.

The tissue into which darn suture is placed during the novel AVWD procedure described herein is the ATFP, or *white line*, a fibrous thickening that consists of parietal fascia from the surrounding pubococcygeus and iliococcygeus portions of the levator ani and the obturator internus muscles [6]. The ATFP is important in providing support to pelvic structures. Cadaveric studies have shown that the anterior segment of the ATFP is attached to the lower posterior side of the body of the pubic bone, approximately 1 cm from the pubic symphysis; that the first 6 cm is attached anteriorly to the anterolateral vagina, creating the anteriorlateral vaginal sulci; and that it is also attached to the ischial spines and gets some fibres from adjacent fascias [29]. Detachment of this lateral support is the primary cause of paravaginal defects and can lead to prolapse of the anterior vaginal wall [30]. Although it may be injured during pregnancy, the ATFP is a point of attachment for many gynaecologic procedures.

However, as in hernia repair, tissue repairs are associated with a high risk of recurrence. Failed tissue repair is most often due to the apposition and suturing together under tension of structures in positions other than normal anatomic, as occurs in traditional anterior colporrhaphy. The principle behind AVWD for the repair of prolapse is similar to that of the nylon darn method, which was commonly used for the treatment of inguinal hernia before the advent of mesh and is still used by some general surgeons instead of mesh repair [31]. The rational for the darn procedure is that it forms a meshwork of non-absorbable sutures that is well tolerated by the tissues and fills the interstices with fibrous connective tissue, providing a buttress across the weakened area of the anterior vaginal wall. This technique is therefore a compensatory repair that facilitates the repair of anterior vaginal wall prolapse without distorting the normal anatomy and without creating suture-line tension, which can be used for central, lateral and combined defects. The procedure is in harmony with the anatomical structures and creates a hammock that reinforces the native support tissue, does not cause tension and confers very a low risk of vaginal mucosal erosion and urinary bladder injury.

The AVWD procedure also creates support under the bladder neck, and can therefore help to alleviate urethral hypermobility. Our postoperative 1-year Q-tip test results showed that average urethral angle dropped from 28.7 ± 6.2 degrees to 15.3 ± 10.4 degrees (p < 0.018). CST was negative in 90.9% of patients. We attribute this results to darn sutures passing under the bladder neck.

## Conclusions

In the present initial series, early postoperative findings indicate that stage II-III anterior POP was successfully treated with the AVWD technique and that the complication rate was low; however, based on the early postoperative appearance of the anatomic site, AVWD does not appear to be as good as the mesh technique. Nevertheless, AVWD can be easily performed in patients who are concerned about serious adverse effects seen with mesh, such as erosion, mesh shrinkage, bladder erosion, fistula, mesh exposure and infection.

## Abbreviations

ATFP: Arcus tendineus fasciae pelvis; AVWD: Anterior vaginal wall darn; CST: Cough stress test; IIQ-7: Seven-item incontinence impact questionnaire; POP: Pelvic organ prolapse; POPQ: POP Quantification; UDI-6: Six-item urogenital distress inventory.

## Competing interests

The authors declare that they have no competing interests.

## Authors’ contributions

The project was developed by OK and ÖA. The clinical database of the patients was acquired by OK, HSS, SB, HA. The manuscript was written by OK, HSS, and ÖA. ŞK performed the statistical analyses. The operative procedures were performed by OK. All authors read and approved the final manuscript.

## Pre-publication history

The pre-publication history for this paper can be accessed here:

http://www.biomedcentral.com/1471-2490/14/51/prepub
